# Construction for the predictive model of quality of life in patients after robot-assisted radical prostatectomy: a cohort study

**DOI:** 10.7150/ijms.100845

**Published:** 2024-11-11

**Authors:** Yuwei Wu, Jinbao Wang, Xianghong Zhou, Wenjie Zhu, Xingyang Su, Bin Zeng, Shuyi Zhang, Xinyue Liu, Zilong Zhang, Yuming Jin, Jiakun Li, Yimao Sun, Linghui Deng, Lu Yang, Yige Bao, Zhenhua Liu, Qiang Wei, Shi Qiu

**Affiliations:** 1 Department of Urology, Institute of Urology and Center of Biomedical Big Data, West China Hospital, Sichuan University, Chengdu, Sichuan 610041, China.; 2 West China Hospital of Sichuan University, No. 37, Guoxue Lane, Chengdu, 610041, Sichuan Province, China.; 3 National Clinical Research Center of Geriatrics, the Center of Gerontology and Geriatrics, West China Hospital, Sichuan University, Chengdu, Sichuan 610041, China.; 4 College of Computer Science, Sichuan University, Chengdu 610065, China.

**Keywords:** robotic-assisted radical prostatectomy, urinary incontinence, quality of life, predictive model, sexual function.

## Abstract

**Background:** Urinary incontinence (UI) and erectile dysfunction (ED) often arise as frequent postoperative complications following robotic-assisted radical prostatectomy (RARP) for prostate cancer (PCa). These issues can significantly diminish patients' quality of life (QoL). The assessment of QoL is even more important because treatment decisions may be influenced by the expected QoL. Few studies have integrated the clinical profiles of patients with magnetic resonance imaging (MRI) metrics to assess postoperative UI and ED.

**Methods:** PCa patients treated with RARP between January 2018 and September 2022 were enrolled in this study. Preoperative clinical baseline characteristics and MRI parameters were retrospectively collected. The Expanded Prostate Cancer Index Composite Short Form (EPIC-26) questionnaire was completed to assess urinary continence and sexual function at regular postoperative follow-up. Preoperative baseline clinical characteristics and MRI parameters were subsequently used to screen for predictors of urinary continence and sexual function after RARP, and predictive models were constructed.

**Results:** A total of 627 patients with PCa who met the criteria were ultimately included in this study, with 1059 follow-up questionnaires. The predictive model for postoperative urinary continence was constructed with respect to age, history of transurethral resection of the prostate (TURP) surgery, clinical T stage (cT), Gleason score (GS), Charlson score, membranous urethral length (MUL), pubic symphysis-prostate apex length (PAL), urethral width, right anal sphincter thickness and anal levator muscle thickness (axial plane). Moreover, body mass index (BMI), cT, age, GS, Charlson score, internal obturator muscle thickness, urethral width and anal sphincter thickness were predictors of short-term and long-term postoperative sexual function. We were able to develop highly effective predictive models for postoperative urinary continence and sexual function in RARP patients by incorporating baseline clinical features and MRI parameters.

**Conclusions:** The predictive model enables the assessment of postoperative urinary continence and sexual function in patients after RARP and offers clinical guidance.

## Introduction

Prostate cancer (PCa) is globally recognized as the second most prevalent malignant neoplasm, with its mortality ranking fifth among all malignancies affecting males. China contributes to 8.2% of the global incidence of PCa annually and accounts for 13.6% of the worldwide mortality associated with this disease^1^. Although the overall incidence of PCa in China is lower than the global average, there has been a notable upwards trend in incidence in recent years^2^. Several studies have indicated that PCa imposes a substantial health burden on the Chinese population^3^. Therefore, it is imperative to implement effective strategies aimed at improving the quality of life of the populace.

Treatment options for PCa include radical prostatectomy (RP), radiotherapy with or without androgen deprivation therapy (ADT), or active surveillance (AS), which are selected on the basis of the risk of PCa recurrence and life expectancy^4^. In addition, RP is a pivotal therapeutic intervention for PCa. Robot-assisted radical prostatectomy (RARP) is widely acknowledged for its superior oncological and functional outcomes owing to its enhanced visual field and heightened precision^5^. Nevertheless, postoperative complications, including urinary incontinence (UI), erectile dysfunction (ED) and others, continue to be frequently observed. All these factors lead to a decrease in patient satisfaction with the surgical procedure and overall quality of life (QoL)^6^.

PCa is characterized by its relatively long natural history, and a significant proportion of patients experience mortality due to factors unrelated to the disease^7^. Therefore, QoL assessment becomes even more critical in situations where treatment decisions may be influenced by anticipated QoL outcomes^8^. The Expanded Prostate Cancer Index Composite (EPIC) is the most appropriate cancer-specific measure for assessing quality of life in urological patients^9^. The concise version of the EPIC-26, comprising 26 items, is the predominant self-assessment scale currently used. It assesses patients' QoL across five aspects: UI, symptoms related to urethral irritation and obstruction, intestinal function, sexual function, and hormone levels. This finding has also been corroborated in Chinese studies^10^.

Previous studies have demonstrated that advanced age, elevated body mass index (BMI), and comorbidities may be associated with an increased risk of UI^11^. Moreover, advanced age, comorbidities, nerve-sparing status, and preoperative erectile function are known factors associated with ED^12^. With the application of magnetic resonance imaging (MRI) technology, the correlation between patient anatomy and QoL has been further investigated. The findings of previous studies have indicated that the morphological characteristics of the prostatic apex and membranous urethral length (MUL) are factors associated with post-RARP UI within a one-year follow-up period^13^. However, limited research has integrated the clinical characteristics of patients with MRI parameters to investigate risk factors for ED.

Given the significance and research status of QoL after RARP, this study aims to explore related risk factors for postoperative urinary continence and sexual function in patients after RARP. The predictive models were constructed on the basis of patients' clinical baseline characteristics and preoperative MRI parameters.

## Methods

### Study population

The study enrolled a total of 627 patients with early prostate cancer who underwent RARP within the West China Prostate Cancer Cohort from January 2018 to September 2022^14^. In addition, all patients included in the cohort underwent appropriate nerve sparing.

The inclusion criteria were as follows: (1) Patients who were diagnosed with PCa and underwent RARP performed by an exceptionally proficient surgeon. The exclusion criteria were as follows: (1) prostate MRI data were unavailable. (2) Relevant follow-up data were either unavailable or incomplete. Approval of the study protocols used was provided by the ethical committee, and the study was registered with the Chinese Clinical Trial Registry. This work is in line with the STROCSS criteria^15^.

### Clinical characteristics

The clinical characteristics of the patients were collected through the hospital information system. Specifically, this information includes patient age, height, weight, comorbidities, preoperative prostate-specific antigen (PSA), history of transurethral resection of the prostate (TURP), Gleason score (GS), clinical T stage (cT), pathological needle biopsy results, etc., at the time of surgical intervention.

BMI was calculated by dividing their weight (in kg) by the square of their height (in m). The Charlson score was calculated on the basis of the patient's medical history and diagnosed comorbidities. The criterion is the sum of corresponding scores of each comorbidity: 1 point: myocardial infarction, congestive heart failure, peripheral vascular disease, cerebrovascular disease (excluding hemiparesis), dementia, chronic lung disease, connective tissue disease, peptic ulcer, mild liver disease, and diabetes mellitus (without complications). 2 points: Diabetes mellitus with end-organ damage, hemiplegia (or paraplegia), moderate to severe renal abnormalities, nonmetastatic solid tumors, leukemia, lymphoma, and multiple myeloma. 3 points: moderate to severe liver function abnormalities. 6 points: metastatic solid tumor, and AIDS. After the cumulative scores were computed, patients were categorized into four groups on the basis of their scores: 0 points, 1-2 points, 3-4 points, and ≥5 points^16^.

### MRI characteristics

**Figure 1:**
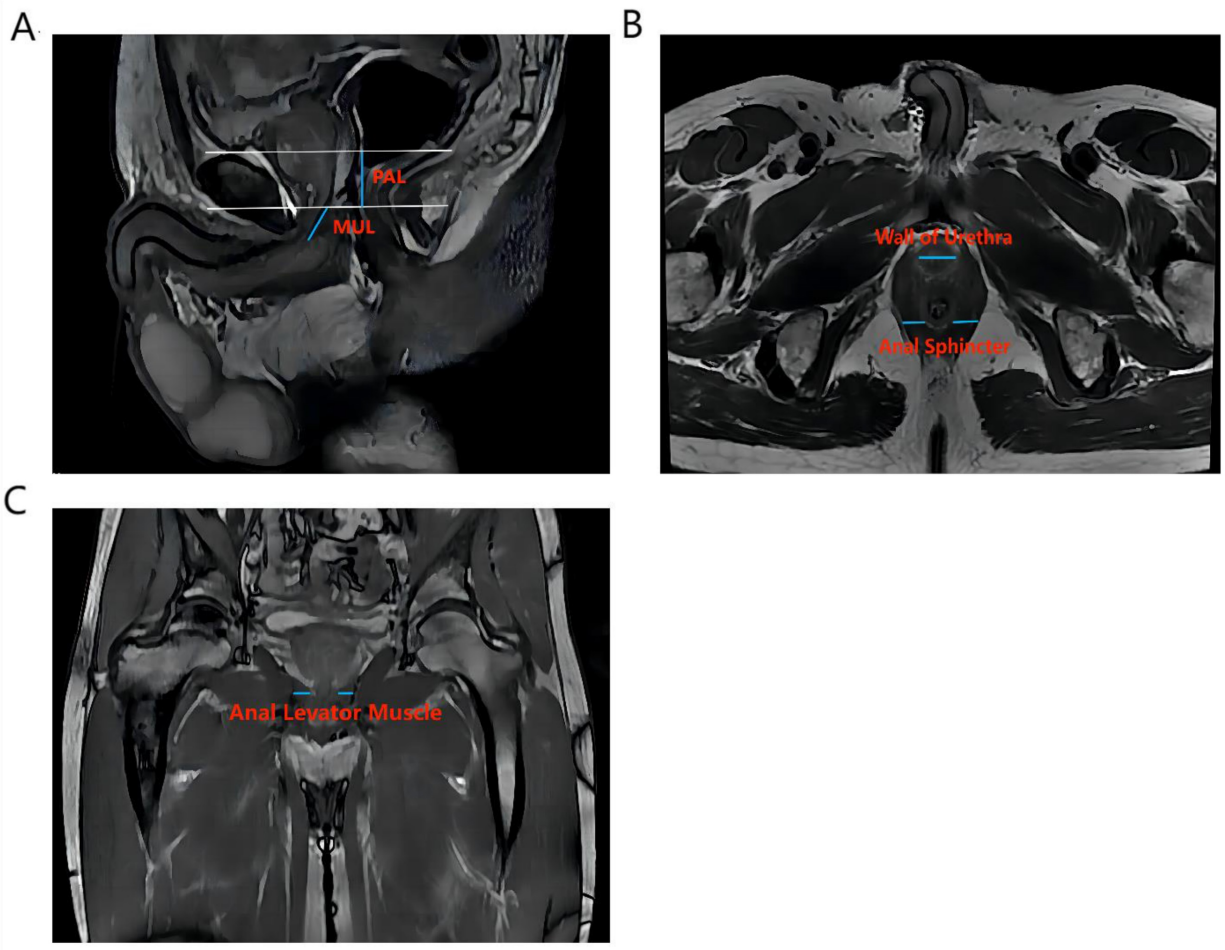
The main measurement indicators of anatomical structure on all T2-weighted images of patients. Figure 1A shows an example of a sagittal parameter on preoperative MR image; Figure 1B shows an example of a preoperative axial plane parameter; Figure 1C shows an example of a preoperative coronal parameter. PAL: pubic symphysis‒prostate apex length; MUL: membranous urethral length

Patients underwent MRI examination via a 3.0T device (GE Company, USA). Measurements of various MRI parameters were conducted on all T2-weighted images of patients who underwent preoperative prostate MRI examinations, with the participation of three observers, including the authors, under the supervision of experienced imaging physicians. The agreement among the three observers was evaluated via the intraclass correlation coefficient (ICC), and the results are presented in Supplementary Table 4. The main measurement indicators are as follows:

Vertical plane (Figure 1A): membrane urethra length (MUL), membrane urethra angle, prostate length, pubic symphysis-prostate apex length (PAL).

Axial plane (Figure 1B): Thickness of the left/right musculus obturator internus, prostate height, prostate width, distance between the outer/inner edges of the anal levator muscle, transverse membranous urethra thickness, left/right anal sphincter thickness, and thickness of the urethral wall.

Coronal plane (Figure 1C): left/right anal levator muscle thickness

Calculation: Anal levator muscle thickness (axial plane) = (outer edge spacing of the anal levator muscle - inner edge spacing of the anal levator muscle)/2. The surface area of the membrane urethra section = (1/2 × thickness of transverse membrane urethra) × (1/2 × thickness of anterior and posterior membrane urethra) × π. Prostate volume = prostate height × prostate length × prostate width ×π/6. Membrane urethra volume = (transverse membrane urethral thickness × 1/2) × (anterior and posterior membrane urethral thickness × 1/2) ×π× membrane urethral length.

### Follow-up data

The assessment of patient QoL and completion of the EPIC-26 scale were implemented during the follow-up period through a combination of outpatient visits and telephone consultations. The scale comprises five dimensions, namely, the urinary incontinence dimension (4 items), the symptoms of urethral stimulation and obstruction dimension (4 items), the intestinal function dimension (6 items), the sexual function dimension (5 items), and the hormone level dimension (5 items). The quality of life in each dimension was assessed on a scale ranging from 0 to 100, with higher scores indicating superior levels^10^.

### Construction of the predictive model

Continuous variables are expressed as the means ± standard deviations (SDs), whereas categorical variables are presented as rates and percentages. P values were obtained via t tests to detect differences among continuous variables and chi-square tests for differences among categorical variables. Univariate analysis was used to assess the correlation between various variables and urinary continence and sexual function outcomes in PCa patients separately, and multivariate analysis was also performed. The results are presented as odds ratios (ORs) and 95% confidence intervals (CIs), and two-sided P values < 0.05 were considered to indicate statistical significance. In addition, predictive factors were also screened by least absolute shrinkage and selection operator (LASSO) regression analysis.

We developed a logistic regression prediction model for urinary continence and linear regression prediction models for sexual function by integrating potential predictors identified through univariate and multivariate analyses, as well as LASSO regression analysis. When the predictive model was constructed, a multivariate score polynomial check and transformation were performed on the continuous variables. The multiple-score polynomial model that best predicts outcome was selected, pairwise interaction terms among independent variables were screened for, and bootstrap resampling was conducted for internal validation (100 resamples).

For the predictive model of urinary continence, we constructed a receiver operating characteristic (ROC) curve on the basis of the results of logistic regression analysis, and the area under the ROC curve (AUC) was used to quantify the discriminative capacity of the model. Moreover, a model calibration curve was employed to evaluate its calibration ability. In addition, for the prediction model of sexual function, the results of linear regression analysis were used to conduct error grid analysis (EGA), and the Parkes EGA was graphed to illustrate the concordance between the predicted values derived from the predictive model and the actual observed values.

The EGA represents the level of error between the predicted and actual values in regions A to E. The results from region A indicate that the predicted and observed values are within a 20% margin, which does not impact clinical decision making. The level of error in Region B exceeds 20%, yet it does not significantly impact clinical decision-making. The discrepancy between the predicted and observed values in region C suggests an overestimation, whereas in region E, it indicates an underestimation. Both types of discrepancy can significantly impact clinical decision-making. Region D indicates undiscovered observations that may influence clinical decision-making^17^-^19^. The model is considered to have great predictive power when 99% of the predicted values fall within Regions A and B^20^.

### Statistical software

The statistical analyses in this study were conducted via EmpowerStats software (http://www. empowerstats.com, X&Y Solutions, Inc., Boston, MA) and the R programming language. The statistical significance of differences was determined by P values, with a threshold set at P <0.05.

## Results

### Construction of a predictive model for urinary continence

On the basis of the inclusion criteria and exclusion criteria, 627 individuals were enrolled in this study. Among these patients, 544 individuals achieved recovery from urinary continence, whereas 83 individuals still used urinal pads. The median follow-up durations were 20.29 months and 25.38 months in the respective populations. The mean age of patients who achieved recovery from urinary continence was 66.87 years, whereas the mean age of patients who did not regain urinary continence was 69.96 years. Compared with individuals with persistent UI, those with restored urinary control presented a relatively younger age profile (66.87±7.44 vs 69.96±7.33, P<0.001), a longer MUL (1.07±0.35 vs 0.97±0.31, P=0.016), a shorter PAL (3.07±0.66 vs 3.28±0.70, P=0.008), and a thinner right anal sphincter. The proportion of individuals without a history of TURP surgery who achieved urinary control recovery was significantly greater than that of those who did not (94.67% vs 85.54%, P=0.005) (Table 1).

The variables in Table 1 were first subjected to univariate analysis. The results demonstrated that age (OR 0.94, 95% CI 0.91-0.97, P<0.001), PAL (OR 0.62, 95% CI 0.44-0.89, P=0.008), right anal sphincter thickness (OR 0.27, 95% CI 0.09-0.89, P=0.031), history of TURP surgery=1 time (OR 0.32, 95% CI 0.16-0.66, P=0.002) and GS=9 score (OR 0.27, 95% CI 0.10-0.72, P=0.009) were significantly associated with recovery from urinary continence in the entire study population. Moreover, MUL was an independent risk factor for UI after RARP (OR 2.52, 95% CI 1.19-5.36; P=0.016). After further multivariate analysis, recovery from urinary continence was not significantly correlated with PAL, whereas the significance of other variables remained unchanged (Table 2 and Supplementary Table 1).

LASSO regression analysis was subsequently conducted on all the variables. When determining the optimal λ value that minimizes average cross-validation error, the identified predictive factors include age, history of TURP surgery=1 time, cT=T2c, cT=T4, GS=6 points, GS=9 points, Charlson score=3--4, MUL, PAL, urethral width, right anal sphincter thickness and anal levator muscle thickness (axial plane).

**Figure 2:**
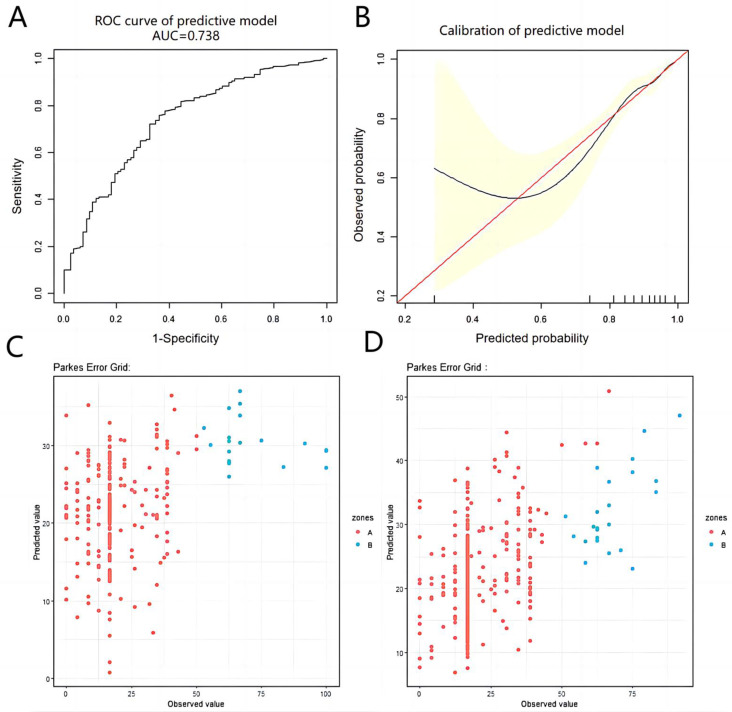
The predictive and calibrated capacity of the prediction models in this study. Figure 2A shows the ROC curve of the prediction model of urinary continence after RARP. Figure 2B shows the calibration curve of the prediction model of urinary continence after RARP. Figure 2C shows the EGA of the predictive model of short-term sexual function score after RARP. Figure 2D shows the EGA of the predictive model of short-term sexual function score after RARP.

The predictive variables obtained through the aforementioned methods were subjected to logistic regression analysis to construct a predictive model. The final formula was 9.34575 - 0.74435* (history of TURP surgery=1 time) + 0.04793*(cT<T2c) + 0.44740*(cT=T2c) + 0.22512*(cT=T3b) - 0.07319*Age(year) + 0.47809*(GS=6 points) - 0.21700*(GS=8 points) - 0.84119*(GS=9 points) + 0.09937*(Charlson score=0) + 0.57344*(Charlson score=3-4 points) + 0.98297*MUL(cm) - 0.13776*PAL(cm) - 0.80022*Urethral width(cm) - 1.61330*Right anal sphincter thickness(cm) - 1.21959*Anal levator muscle thickness (axial plane)(cm). The AUC of the ROC curve for the obtained model was 0.738 (>0.7), indicating excellent predictive capacity (Figure 2A). However, in the calibration curve, some of the predicted values are underestimated compared with the actual values; nevertheless, the overall calibration capability remains reasonable (Figure 2B).

### Construction of a predictive model for sexual function

The entire study population was enrolled, 266 of which were short-term follow-up patients, while the other 437 were long-term follow-up patients, with a cut-off of 12 months after surgery. The mean age of the short-term follow-up population was 66.92 years, with a mean BMI of 24.02 and an average follow-up duration of 6.34 months, and it was 67.51 years in the long-term follow-up population, with a mean BMI of 24.17 and an average follow-up duration of 32.13 months. Details of the various indicators of the patients are shown in Supplementary Table 2.

In the short-term follow-up population, univariate analysis revealed significant differences in age, BMI, cT=T4, GS=9 points, left internal obturator muscle thickness and urethral width. However, only age (β=-0.45, 95% CI=-0.73--0.18, P=0.001) and cT=T4 (β=-10.31, 95% CI=-19.90--0.72, P=0.036) were found to be statistically significant in the multivariate analysis, suggesting that age and cT=T4 were potential predictors of sexual function in the short term following RARP (Table 3 and Supplementary Table 3). A 1-year increase in age was associated with a 0.45-point decrease in the sexual function score. All variables were subsequently subjected to LASSO regression analysis, which revealed that the predictive factors for sexual function included age, BMI, history of TURP surgery=1 time, cT=T2c, cT=T3a, cT=T4, GS=6, GS=9, Charlson score=0, left internal obturator muscle thickness, urethral width and right anal sphincter thickness. By utilizing potential predictor variables obtained through the aforementioned approaches, we have derived final predictive model formulas. In the short-term follow-up population, the formula was 8.51449 + 0.59933 * BMI (kg/m^2^) - 3.64065 * (history of TURP surgery = 1 time) + 7.27015 * (cT <T2c) + 10.59556 * (cT=T2c) + 3.20239 * (cT=T3b) - 3.46238 * (cT=T4) - 0.29899 * Age (years) + 0.04724 * (GS=6 scores) - 2.99144 * (GS=7 scores) - 5.62285 * (GS=9 scores) + 3. 15437 * (Charlson score=0 scores) - 1.70589 * (Charlson score=3-4 scores) + 6.21213 * Left internal obturator muscle thickness (cm) + 5.33641 * Urethral width (cm) - 6.38418 * Right anal sphincter thickness (cm). The EGA of the prediction model is illustrated in Figure 2C. The results indicated that all the predicted values were situated within either region A or B, with 92.9% falling within region A and 7.1% falling within region B.

In the long-term follow-up population, univariate analysis revealed that age, baseline PSA, cT=T2c, cT=T3b, GS=7,8,9 points, Charlson score=1-2 points, PAL, left/right internal obturator muscle thickness, urethral width and left anal sphincter thickness were associated with sexual function scores in long-term QoL following RARP. A multivariate analysis revealed that age, cT=T2c, GS=7,8,9, Charlson score=1-2, Charlson score ≥ 5 and left anal sphincter thickness were significantly correlated with sexual function scores (Table 3 and Supplementary Table 3). All variables were subsequently subjected to LASSO regression analysis, which revealed that age, BMI, GS=6 points, GS=8 points, baseline PSA, cT=T2c, cT=T3b, history of TURP surgery=0, Charlson score=1-2 points, PAL, left internal obturator muscle thickness, urethral width, left/right anal sphincter thickness, urethral wall thickness, left anal levator muscle thickness (coronal plane), prostate volume and membranous urethra volume were potential predictors of sexual function scores. By utilizing potential predictor variables obtained through the aforementioned approaches, we derived a final predictive model formula: 10.09756 + 0.17601*BMI (kg/m2) - 0.00928*Baseline PSA (ng/ml) -1.23761*(cT <T2c) - 4.44128*(cT=T2c) + 0.02478*(cT=T3a) - 4.81480*(cT=T3b) + 8.34815*((age (year)/100) ^ 2) + 6.33283*(GS=6 points) + 0.29983*(GS=8 points) - 4.81519*(GS=9 points) + 3.39984*(Charlson score=0 points) + 0.55016*(Charlson score=3-4 points) - 1.27232*PAL (cm) + 2.85673*Left internal obturator muscle thickness (cm) -1.79791 * Right internal obturator muscle thickness (cm) + 4.91769*urethral width (cm) - 12.37772*Left anal sphincter thickness (cm) -2. 76090*right anal sphincter thickness (cm) - 4.16998*urethral wall thickness (cm) + 0.92614 * left anal levator muscle thickness (coronal plane) (cm) ^ -2) - 0.03124 * prostate volume (cm^3^) - 1.34870*membranous urethra volume (cm^3^). The EGA of the model revealed that the predicted values are distributed within regions A and B, with 94.5% falling within region A and the remaining 5.5% falling within region B (Figure 2D). The constructed prediction model exhibited excellent capacity for accurate predictions.

## Discussion

The present study aimed to investigate postoperative urinary continence and sexual function outcomes following RARP and subsequently developed and validated predictive models. The results indicated that age, MUL, and PAL were significant predictors of urinary continence recovery after RARP. The probability of postoperative urinary continence recovery is inversely associated with age and PAL, whereas the magnitude of MUL is directly proportional. A prospective study conducted by Stanford *et al.* involving over 1200 men who underwent radical prostatectomy (RP) demonstrated a significant association between age and the rate of recovery from urinary continence. Specifically, patients younger than 60 years of age are more likely to recover from UI^21^. Multiple studies have incorporated MUL into predictive models for UI^22^-^24^, demonstrating an inverse relationship between the length of MUL and the duration of postoperative recovery required for urinary function^24^. Notably, patients who achieved urinary continence at 3 months postsurgery had shorter PAL lengths than did those who experienced continence issues (26.0 vs 28.1 mm, P<0.05)^25^. The results of these studies are in line with the findings of this study.

The present study also revealed that GS, anal sphincter thickness and history of TURP surgery were significant predictive factors for recovery from urinary continence following RARP. These factors have been documented in the literature, and the findings are consistent with those of this study. Tienza *et al.* reported that a patient's surgical history of TURP was an independent prognostic factor for postoperative UI (OR 6.13, CI 95% 1.86-20.18, P=0.003)^26^. Gupta *et al.* also reported a greater prevalence of UI among patients who underwent TURP than among those without prior TURP surgery^27^. However, with increasing age, the risk of both benign prostatic hyperplasia (BPH) and prostate cancer (PCa) increases. Concurrently, there is a growing trend in the number of patients with a history of TURP who undergo RARP^27^. Consequently, delineating the precise association between TURP surgery history and urinary control poses a significant challenge. Additionally, a retrospective study conducted by Palisaar *et al.*, involving 4,028 patients, revealed that both cT and GS emerged as independent predictors of UI following RARP^28^.

Variable selection is a crucial issue in the development of prediction models, as the predictive performance of models is partially influenced by the variables incorporated in the models. In this study, we employed LASSO regression, a machine learning technique widely used for variable selection in academic research^29^. It imposes a penalty on the absolute size of regression coefficients by considering the tuning parameter λ. Consequently, LASSO regression effectively drives the coefficients of irrelevant variables toward zero, thereby facilitating automatic variable selection and promoting the interpretability of the model. Moreover, LASSO regression frequently avoids overfitting and results in better prediction performance than the random forest algorithm in sparse datasets^30^.

For the constructed predictive model of urinary continence, this study integrated preoperative clinical baseline characteristics and MRI features. The AUC of the predictive model exceeded 0.7, indicating excellent predictive ability. Furthermore, the calibration curve demonstrated high levels of calibration and discrimination in the model. By incorporating preoperative clinical parameters, including the American Society of Anesthesiologists (ASA) grade and surgical experience, Collette *et al.* developed a predictive model for urinary continence^31^. Although it encompasses numerous preoperative clinical parameters, the model's AUC merely reached 0.61 or 0.60, with no consideration given to the impact of MRI features. The AUCs of the calibrated models developed by Pinkhasov *et al.* at 6, 12, and 24 months after RARP were 0.52, 0.52, and 0.76, respectively. Notably, the final models exhibited superior predictive power compared with any individual clinical variable^32^. None of the models constructed by these studies incorporated MRI parameters and had relatively low AUCs. Honda *et al.* attempted to develop a novel prediction model for urinary continence^33^, incorporating MUL and lift muscle thickness while disregarding the influence of clinical baseline features despite their inclusion of MRI characteristics. Additionally, a predictive model developed by Miyake *et al.* incorporated only the bladder neck angle and MUL and exhibited unsatisfactory predictive performance^34^. In this context, the present study effectively integrates clinical and MRI features to successfully develop and validate a predictive model for postoperative urinary control, thereby conferring significant advantages.

In terms of sexual function, among the variables ultimately used to construct the predictive model by screening relevant variables of the sexual function dimension score, BMI, cT, age, GS, Charlson score, obturator muscle thickness, urethral width, and anal sphincter thickness were common predictors of postoperative short-term and long-term QoL models. Haskins *et al.* developed a predictive model for postoperative sexual function and reported that men under the age of 60 years, with a normal BMI, without diabetes or hypertension, with nerve-sparing surgery, and with no history of smoking presented an increased likelihood of achieving functional erections following surgery^35^. A study conducted by Neumaier *et al.* also demonstrated a significant association between BMI> 30 kg/m2 (r <0.001) and age (r=0.011) and decreased sexual function after surgery^36^. In this study, univariate analysis revealed a negative correlation between age and short-term as well as long-term sexual function scores, with each one-year increase in age resulting in decreases of 0.5 (r=0.001) and 0.6 (r<0.001) points, respectively, while the multivariate results were 0.4 (r=0.023) and 0.6 (r<0.001) points, respectively. The observed differences, although small in magnitude, were statistically significant. Therefore, the significance of age and BMI in predicting sexual function is evident. In a study involving 643 patients aimed at constructing a nomogram for predicting sexual function one year after RARP, the authors reported a negative correlation between the Gleason score (r=0.0002) and the Charlson score (r=0.02) with sexual function^37^, which aligns with the findings of the present study. The present study also revealed a potential positive correlation between the thickness of the obturator internus muscle and the width of the urethra with respect to sexual function scores. However, this may be attributed to chance, and no relevant literature has been identified to date.

The present study has several notable strengths. This study integrated preoperative clinical baseline characteristics and MRI characteristics of patients undergoing RARP to predict postoperative urinary continence and sexual function, explored relevant predictors, and successfully constructed a predictive model with relatively superior predictive ability. This model enables the anticipation of postoperative urinary control and sexual function prior to RARP, thereby guiding postoperative recovery. However, there is a paucity of research in this specific domain. Furthermore, the prediction models constructed in this study integrated the variables selected through univariate/multivariate analysis and LASSO regression analysis, thereby enhancing their predictive ability. Therefore, this study has significant clinical implications.

This study has several limitations that should be taken into account. First, we excluded patients who underwent surgical procedures performed by a single surgeon. Despite the surgeon's extensive experience, variations in surgical practices inevitably arise. Second, pertinent data on radiotherapy and endocrine therapy were not included, which may have potentially affected the investigation of QoL in this study. The validation of the findings in this study calls for an increased sample size and more comprehensive data in the future.

## Conclusion

Our study suggests that the predictive model incorporating preoperative clinical baseline characteristics and MRI characteristics enables the assessment of postoperative urinary control and sexual function in patients after RARP. These findings could offer valuable insights and clinical guidance for healthcare professionals and patients in the future.

## Supplementary Material

Supplementary tables.

## Figures and Tables

**Scheme 1:**
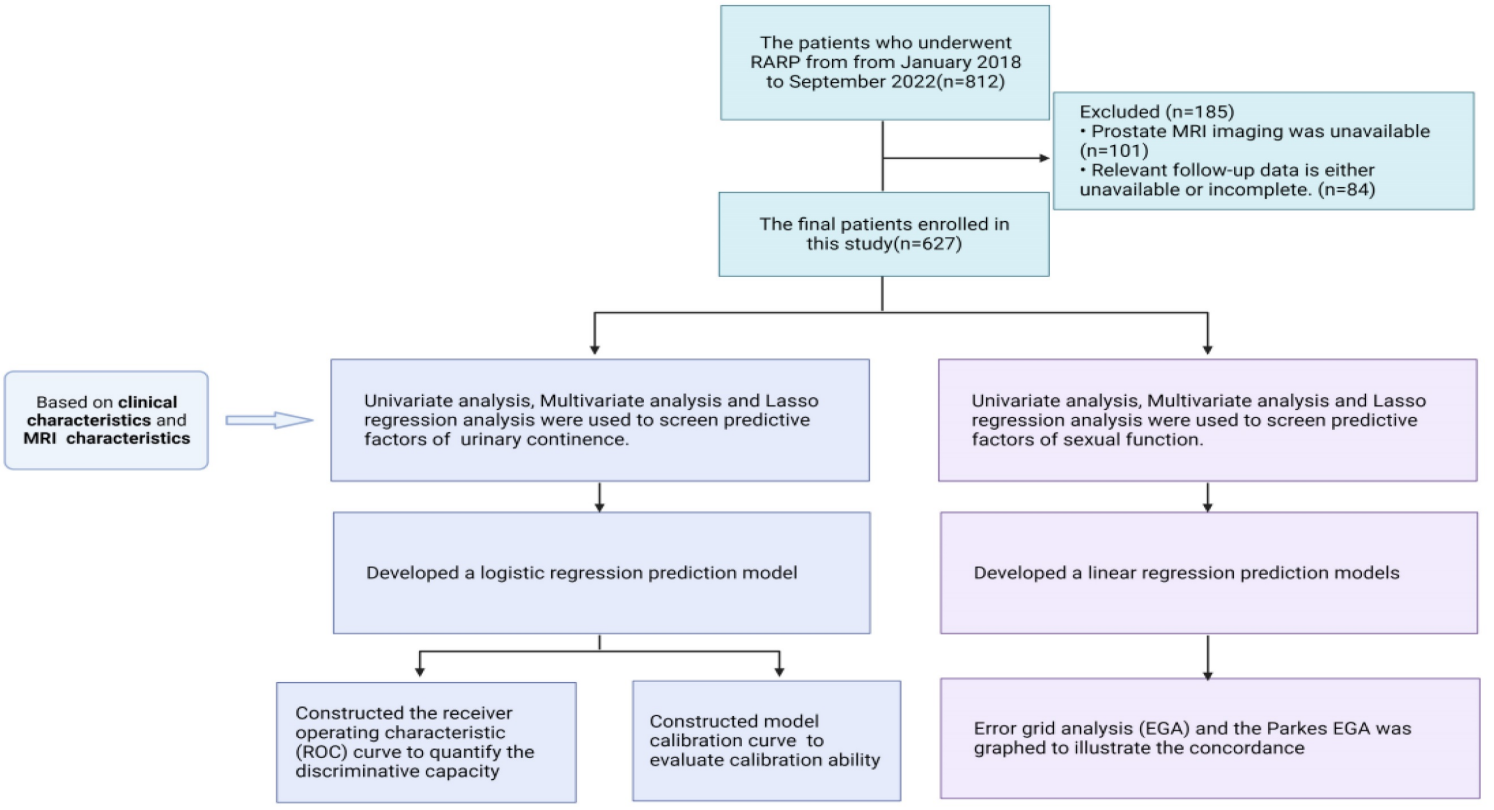
Flow chart of inclusion and exclusion criteria in this study.

**Table 1:** Baseline demographic, clinical and anatomical characteristics of the study patients.

Characteristics	Urinary continence	Urinary incontinence	P value
Size	83	544	
Follow-up time (month)	20.29 ± 18.37	25.38 ± 16.08	0.009
Age (year)	69.96 ± 7.33	66.87 ± 7.44	<0.001
BMI (kg/m^2^)	24.33 ± 2.34	24.11 ± 2.70	0.483
PSA (ng/mL)	26.22 ± 48.51	23.07 ± 40.62	0.522
Urethral width (cm)	1.11 ± 0.29	1.07 ± 0.20	0.181
Urethral wall thickness (cm)	1.08 ± 0.16	1.07 ± 0.17	0.804
Membranous urethral length (cm)	0.97 ± 0.31	1.07 ± 0.35	0.016
Membranous urethral angle (°)	122.42 ± 11.72	120.83 ± 10.48	0.207
Transverse width of the membranous urethra(cm)	1.12 ± 0.18	1.10 ± 0.22	0.426
fore-and-aft width of the membranous urethra(cm)	1.11 ± 0.22	1.09 ± 0.22	0.598
Sectional area of membranous urethra (cm^2^)	0.99 ± 0.33	0.97 ± 0.37	0.557
Membranous urethra volume (cm^3^)	0.94 ± 0.39	1.02 ± 0.47	0.169
Left internal obturator muscle thickness (cm)	1.81 ± 0.30	1.84 ± 0.31	0.402
Right internal obturator muscle thickness (cm)	1.82 ± 0.31	1.87 ± 0.32	0.192
Left anal levator muscle thickness (coronal plane) (cm)	0.96 ± 0.24	0.93 ± 0.23	0.411
Right anal levator muscle thickness (coronal plane) (cm)	0.97 ± 0.23	0.95 ± 0.23	0.454
Anal levator muscle thickness (axial plane) (cm)	1.09 ± 0.20	1.05 ± 0.22	0.078
Left anal sphincter thickness(cm)	0.54 ± 0.14	0.54 ± 0.17	0.946
Right anal sphincter thickness (cm)	0.58 ± 0.32	0.53 ± 0.15	0.011
Pubic symphysis-prostate apex length (cm)	3.28 ± 0.70	3.07 ± 0.66	0.008
Prostate volume (cm^3^)	31.55 ± 14.99	32.26 ± 18.46	0.740
No. preoperative cT stage (%)			0.545
<T2c	50 (60.24%)	318 (58.46%)	
T2c	16 (19.28%)	138 (25.37%)	
T3a	5 (6.02%)	25 (4.60%)	
T3b	6 (7.23%)	41 (7.54%)	
T4	6 (7.23%)	22 (4.04%)	
No. pathological GS (%)			0.069
6	6 (7.23%)	82 (15.07%)	
7	45 (54.22%)	319 (58.64%)	
8	14 (16.87%)	75 (13.79%)	
9	18 (21.69%)	67 (12.32%)	
10	0 (0.00%)	1 (0.18%)	
No. Charlson score (%)			0.482
0	34 (40.96%)	220 (40.44%)	
1-2	40 (48.19%)	255 (46.88%)	
3-4	6 (7.23%)	60 (11.03%)	
≥5	3 (3.61%)	9 (1.65%)	
No. history of TURP surgery (%)			0.005
0	71 (85.54%)	515 (94.67%)	
1	12 (14.46%)	28 (5.15%)	
3	0 (0.00%)	1 (0.18%)	

Note: The results in the table are Middle + SD/N (%)

**Table 2:** Univariate and multivariate analyses of anatomical factors considered in the predictive model of urinary continence.

Characteristics	Univariate analysis	P value	Multivariate analysis	P value
Membranous urethral length (cm)	2.52 (1.19, 5.36)	0.016	2.73 (1.18, 6.36)	0.020
Pubic symphysis-prostate apex length (cm)	0.62 (0.44, 0.89)	0.008	0.88 (0.59, 1.32)	0.547
Right anal sphincter thickness(cm)	0.27 (0.09, 0.89)	0.031	0.15 (0.04, 0.53)	0.004

Note: The results in the table are ORs (95% CIs) and P values.

**Table 3:** Univariate and multivariate analyses of anatomical factors considered in the predictive model of sexual function.

Characteristics	Univariate analysis	P value	Multivariate analysis	P value
Short-term after RARP (≤12 months)				
Left internal obturator muscle thickness(cm)	11.28 (4.18, 18.38)	0.002	5.29 (-2.57, 13.16)	0.188
Urethral width (cm)	11.62 (2.41, 20.83)	0.014	6.54 (-3.03, 16.12)	0.182
Long-term after RARP (>12 months)				
Urethral width (cm)	6.40 (0.10, 12.70)	0.047	0.90 (-5.07, 6.86)	0.769
Left internal obturator muscle thickness(cm)	6.27 (1.92, 10.63)	0.005	3.03 (-2.39, 8.45)	0.273
Right internal obturator muscle thickness(cm)	4.13 (0.03, 8.22)	0.049	-2.99 (-8.17, 2.18)	0.258
Left anal sphincter thickness(cm)	-9.39 (-17.41, -1.36)	0.022	-13.07 (-20.53, -5.62)	<0.001
Pubic symphysis-prostate apex length (cm)	-2.49 (-4.45, -0.54)	0.013	-0.94 (-2.86, 0.98)	0.340

Note: The results in the table are OR (95% CI) P values. The population was considered a short-term follow-up population and long-term follow-up population on the basis of a cut-off of 12 months after RARP.

## Abbreviations

UI: Urinary incontinence
ED: Erectile dysfunction
RP: Radical prostatectomy
RARP: Robotic-assisted radical prostatectomy
PCa: prostate cancer
QoL: Quality of life
MRI: Magnetic resonance imaging
TURP: Transurethral resection of the prostate
cT: Clinical T-stage
GS: Gleason score
MUL: Membranous urethral length
PAL: Pubic symphysis-prostate apex length
BMI: Body mass index
PSA: Prostate-specific antigen
SD: Standard deviation
OR: Odds ratio
CI: Confidence interval
Lasso: Least absolute shrinkage and selection operator
AUC: Area under the ROC curve
EGA: Error grid analysis
ASA: American Society of Anesthesiologists
