# Supplementary material for: Construction for the predictive model of quality of life in patients after robot-assisted radical prostatectomy: a cohort study
Source: Int J Med Sci. 2024 Nov 11;21(15):2981–91. doi: 10.7150/ijms.100845 (PMC11610334; doi:10.7150/ijms.100845)
Supplement: Supplementary file 1 — Supplementary tables. [file ijmsv21p2981s1.pdf]

Supplementary Table 1. Univariate and multivariate analyses of clinical factors considered in the predictive model of urinary continence

Characteristics	Univariate analysis	P value	Multivariate	P value
Age (year)	0.94 (0.91, 0.97)	**<0.001**	0.94 (0.91, 0.98)	**0.001**
History of TURP surgery (time)				
0	1		1	
1	0.32 (0.16, 0.66)	**0.002**	0.43 (0.20, 0.93)	**0.032**
3	107428.36(0.00, Inf)	0.983	184754.43(0.00, Inf)	0.989
Gleason score				
6	1		1	
7	0.52 (0.21, 1.26)	0.146	0.64 (0.26, 1.59)	0.339
8	0.39 (0.14, 1.07)	0.068	0.48 (0.17, 1.34)	0.160
9	0.27 (0.10, 0.72)	**0.009**	0.29 (0.11, 0.80)	**0.017**
10	57017.11(0.00, Inf)	0.984	105291.48(0.00, Inf)	0.990

Note: The results in the table are ORs (95% CIs) and P values.

Supplementary Table 2. Baseline demographic, clinical and MRI characteristics of the study patients in the predictive model of sexual function

Characteristics	Short-term after RARP	Long-term after RARP
Age (year)	66.92 ± 7.60	67.51 ± 7.46
Postoperative time(month)	6.34 ± 3.67	32.13 ± 13.42
BMI（kg/m2）	24.02 ± 2.66	24.17 ± 2.69
Baseline PSA（ng/ml）	21.43 ± 37.63	24.55 ± 41.73
Membranous urethral length (cm)	1.05 ± 0.32	1.06 ± 0.37
Membranous urethral angle (°)	120.23 ± 9.76	121.71 ± 10.98
Pubic symphysis-prostate apex length (cm)	3.07 ± 0.63	3.11 ± 0.69
c(cm)	1.89 ± 0.30	1.80 ± 0.31
Right internal obturator muscle thickness	1.91 ± 0.30	1.83 ± 0.33
Urethral width (cm)	1.08 ± 0.23	1.06 ± 0.21
Transverse thickness of the membranous urethra(cm)	1.12 ± 0.24	1.08 ± 0.23
fore-and-aft thickness of the membranous urethra(cm)	1.10 ± 0.21	1.08 ± 0.23
Left anal sphincter thickness(cm)	0.55 ± 0.16	0.53 ± 0.17
Right anal sphincter thickness (cm)	0.57 ± 0.22	0.51 ± 0.15
Urethral wall thickness (cm)	1.05 ± 0.15	1.09 ± 0.17
Left anal levator muscle thickness (coronal plane) (cm)	0.97 ± 0.20	0.92 ± 0.24
Right anal levator muscle thickness (coronal plane) (cm)	0.99 ± 0.22	0.93 ± 0.23
Anal levator muscle thickness (axial plane) (cm)	1.09 ± 0.20	1.02 ± 0.22
Sectional area of membranous urethra	1.00 ± 0.39	0.94 ± 0.38

Prostate volume（cm^3）	33.54 ± 20.07	31.70 ± 16.99
Membranous urethra volume（cm3）	1.04 ± 0.46	0.98 ± 0.48
Sexual function score	22.10 ± 17.70	22.30 ± 14.43
History of TURP surgery (time)		
0	239 (89.85%)	418 (95.65%)
1	26 (9.77%)	19 (4.35%)
3	1（0.38%）	0（0.00%）
clinical T-stage		
<T2c	149 (56.02%)	263 (60.18%)
T2c	71 (26.69%)	100 (22.88%)
T3a	11 (4.14%)	24 (5.49%)
T3b	21 (7.89%)	34 (7.78%)
T4	14 (5.26%)	16 (3.66%)
Gleason score		
6	39 (14.66%)	55 (12.59%)
7	161 (60.53%)	250 (57.21%)
8	30 (11.28%)	70 (16.02%)
9	36 (13.53%)	61 (13.96%)
10	0 (0.00%)	1 (0.23%)
Charlson score		
0	119 (44.74%)	162 (37.07%)
1-2	118 (44.36%)	214 (48.97%)
3-4	25 (9.40%)	52 (11.90%)
≥5	4 (1.50%)	9 (2.06%)

Note: The results in the table are Middle + SD/N (%)

Supplementary Table 3. Univariate and multivariate analyses of clinical factors considered in the predictive model of sexual function

Characteristics	Univariate analysis	P value	Multivariate analysis	P value
**Short-term after RARP (≤12 months)**				
Age (year)	-0.45 (-0.73, -0.18)	**0.001**	-0.33 (-0.63, -0.03)	**0.032**
BMI（kg/m2)	0.90 (0.11, 1.70)	**0.027**	0.52 (-0.30, 1.34)	0.215
clinical T-stage				
T2c	2.18 (-2.76, 7.13)	0.388	3.37 (-1.52, 8.27)	0.178
T3a	-8.79 (-19.51, 1.92)	0.109	-6.21 (-17.00, 4.57)	0.260
T3b	-4.90 (-12.89, 3.10)	0.231	-2.63 (-10.84, 5.59)	0.532
T4	-10.31(-19.90, -0.72)	**0.036**	-8.89 (-19.23, 1.46)	0.093
Gleason score				
6	0		0	
7	-4.29 (-10.44, 1.87)	0.173	-2.40 (-8.55, 3.75)	0.445
8	-5.88 (-14.25, 2.49)	0.170	0.44 (-8.34, 9.21)	0.923
9	-9.96(-17.93, -2.00)	**0.015**	-3.42 (-11.86, 5.02)	0.427
**Long-term after RARP(>12 months)**				
Age (year)	-0.63 (-0.81, -0.46)	**<0.001**	-0.64 (-0.83, -0.46)	**<0.001**
Baseline PSA（ng/ml）	-0.04 (-0.07, -0.00)	**0.032**	-0.01 (-0.04, 0.02)	0.522
clinical T-stage				
<T2c	0		0	
T2c	-5.11 (-8.39, -1.83)	**0.002**	-4.03 (-7.06, -1.00)	**0.010**
T3a	-0.97 (-6.92, 4.99)	0.751	1.12 (-4.48, 6.72)	0.696
T3b	-7.25(-12.35, -2.16)	**0.006**	-3.40 (-8.47, 1.68)	0.190
T4	-1.95 (-9.15, 5.25)	0.595	3.43 (-3.27, 10.13)	0.317
Gleason score				
6	0		0	

7	-8.15(-12.26, -4.03)	**<0.001**	-5.55 (-9.42, -1.69)	**0.005**
8	-9.56(-14.54, -4.58)	**<0.001**	-6.17(-10.92, -1.41)	0.011
9	-12.60(-17.74, -7.46)	**<0.001**	-9.61(-14.74, -4.47)	**<0.001**
10	-13.62(-41.49, 14.25)	0.339	-10.67(-37.08, 15.74)	0.429
Charlson score				
0	0		0	
1-2	-3.31 (-6.23, -0.38)	**0.027**	-3.99 (-6.69, -1.29)	**0.004**
3-4	-4.48 (-8.96, 0.00)	0.051	-3.35 (-7.57, 0.86)	0.120
>5	5.59 (-4.04, 15.21)	0.256	10.93 (2.00, 19.85)	**0.017**

Note: The results in the table are ORs (95% CIs) and P values.
The population was considered a short-term follow-up population and long-term follow-up population on the basis of a cut-off of 12 months after RARP.

Supplementary Table 4. Interobserver agreements for the measurements of various MRI parameters

Imaging features	ICC (95%CI)*	P value
Membrane urethra length	0.800 (0.782,0.818)	<0.001
Prostate length	0.679 (0.652,0.704)	<0.001
Prostate height	0.659 (0.631,0.686)	<0.001
Prostate width	0.619 (0.589,0.648)	<0.001
pubic symphysis-prostate apex length	0.669 (0.642,0.696)	<0.001
Thickness of the left musculus obturator internus	0.727 (0.703,0.749)	<0.001
Thickness of the right musculus obturator internus	0.780 (0.760,0.799)	<0.001
Distance between the outer edges of the anal levator muscle	0.512 (0.477,0.546)	<0.001
Distance between the inner edges of the anal levator muscle	0.709 (0.684,0.733)	<0.001
Transverse membranous urethra thickness	0.561 (0.528,0.593)	<0.001
Left anal sphincter thickness	0.653 (0.625,0.680)	<0.001
Right anal sphincter thickness	0.611 (0.581,0.641)	<0.001
Thickness of urethral wall	0.660 (0.633,0.687)	<0.001
Left anal levator muscle thickness	0.559 (0.526,0.591)	<0.001
Right anal levator muscle thickness	0.618 (0.588,0.647)	<0.001

* Interobserver agreement for continuous variables was assessed with the intraclass correlation coefficient (ICC). Agreement was considered poor (ICC<0.2), fair (ICC: 0.2–0.4), moderate (ICC: 0.4–0.6), substantial (ICC: 0.6–0.8), or excellent (ICC>0.8).
